# 恩沙替尼联合放疗治疗*TPM3-ALK*融合晚期肺原发上皮样炎性肌纤维母细胞肉瘤1例

**DOI:** 10.3779/j.issn.1009-3419.2025.102.40

**Published:** 2025-12-20

**Authors:** Ye ZHAO, Shuangbing XU

**Affiliations:** ^1^430022 武汉，华中科技大学同济医学院附属协和医院肿瘤中心; ^1^Cancer Center, Union Hospital, Tongji Medical College, Huazhong University of Science and Technology, Wuhan 430022, China; ^2^430022 武汉，肿瘤精准放射治疗湖北省重点实验室; ^2^Hubei Key Laboratory of Precision Radiation Oncology, Wuhan 430022, China; ^3^430022 武汉，华中科技大学同济医学院附属协和医院肿瘤放射治疗研究所; ^3^Institute of Radiation Oncology, Union Hospital, Tongji Medical College, Huazhong University of Science and Technology, Wuhan 430022, China

**Keywords:** 肺肿瘤, 肺原发上皮样炎性肌纤维母细胞肉瘤, *ALK*融合, 靶向治疗, 放疗, Lung neoplasms, Primary pulmonary epithelioid inflammatory myofibroblastic sarcoma, Anaplastic lymphoma kinase fusion, Targeted therapy, Radiotherapy

## Abstract

上皮样炎性肌纤维母细胞肉瘤（epithelioid inflammatory myofibroblastic sarcoma, EIMS）是一种罕见且极具侵袭性的间叶来源肿瘤，通常与间变性淋巴瘤激酶（anaplastic lymphoma kinase, *ALK*）基因融合有关。手术是早中期患者主要治疗手段，但晚期患者的治疗策略目前尚无明确共识。肺原发EIMS更为罕见，仅有个案报道。虽然单药ALK-酪氨酸激酶抑制剂（tyrosine kinase inhibitors, TKIs）靶向治疗是一种可行方案，但临床疗效仍不理想。本文报告了1例晚期肺原发EIMS伴有*TPM3-ALK*融合的病例，接受第二代ALK-TKI恩沙替尼一线靶向治疗及残留病灶和转移灶放疗后肿瘤明显缩小并得到持续控制，无进展生存期（progression-free survival, PFS）超过32个月，且未观察到明显治疗相关不良反应。本文探讨了以基因检测为证据的靶向治疗联合局部放疗的可行性，旨在为晚期肺原发EIMS患者提供新的治疗选择。

## Abstract (en)

Epithelioid inflammatory myofibroblastic sarcoma (EIMS) is a rare and highly aggressive mesenchymal neoplasm that is frequently associated with anaplastic lymphoma kinase (*ALK*) gene fusion. Surgical resection remains the cornerstone of treatment for patients with early- and intermediate-stage EIMS; however, a standardized therapeutic approach for advanced-stage EIMS has yet to be established. Primary pulmonary EIMS is exceedingly rare, with only a limited number of cases reported in the literature. While treatment with ALK-tyrosine kinase inhibitors (TKIs) is considered a viable therapeutic option, and clinical outcomes with monotherapy using ALK-TKIs have frequently been suboptimal. This study presents a case of advanced primary pulmonary EIMS with a *TPM3-ALK* fusion. The patient received first-line targeted therapy with the second-generation ALK-TKI Ensartinib, in conjunction with radiotherapy for residual and metastatic lesions. This treatment regimen resulted in significant tumor reduction and sustained disease control. The progression-free survival (PFS) exceeded 32 months, with no significant treatment-related adverse events observed. This study investigates the feasibility of combining targeted therapy with local radiotherapy, guided by genetic testing, to offer novel treatment strategies for patients with advanced primary pulmonary EIMS.

上皮样炎性肌纤维母细胞肉瘤（epithelioid inflammatory myofibroblastic sarcoma, EIMS）是炎性肌纤维母细胞瘤（inflammatory myofibroblastic tumor, IMT）的一种罕见和侵袭性亚型，与经典IMT不同，EIMS预后不良，且更易发生局部复发和快速进展^[1]^。

EIMS并非表现为IMT的梭形细胞形态，其主要由饱满的圆形上皮样肿瘤细胞组成，可见泡状细胞核，黏液样间质富含嗜中性粒细胞浸润。EIMS的免疫组化特点是间变性淋巴瘤激酶（anaplastic lymphoma kinase, ALK）蛋白的弥散性或局灶性表达，通常位于肿瘤细胞的核膜或核周区域。分子检测分析可能揭示特定的基因特征，如有*ALK*融合突变，可进一步支持诊断^[1]^。

EIMS最常见的受累部位是腹腔，尤其是肠系膜和大网膜。肺原发EIMS极为罕见，通常表现为肺实质内的孤立结节或肿块，因病灶位于胸腔，患者可有胸痛、咳嗽、胸闷等呼吸道症状^[2,3]^。EIMS的治疗以手术切除为主，对于存在转移性病灶的患者，需根据肿瘤的范围、组织病理学特征和分子特征，考虑其他治疗方法，如化疗、放疗和靶向治疗。*ALK*融合突变的EIMS患者通常首选第一或二代ALK-酪氨酸激酶抑制剂（tyrosine kinase inhibitors, TKIs），但是无进展生存期（progression-free survival, PFS）一般不超过1年^[2-4]^，仅有极少病例获得3-4年的长程疾病控制^[5,6]^。由于EIMS的罕见性，目前缺乏治疗指南及共识，临床治疗决策需要制定个体化方案。

本文报道了1例原发于肺并伴有*TPM3-ALK*融合突变的晚期EIMS患者，在接受第二代ALK-TKI恩沙替尼一线治疗和残留病灶及转移灶放疗后PFS超过32个月，是目前为止有文献报道的一线缓解持续时间最长的转移性肺原发EIMS病例，为未来探索该疾病的最佳治疗模式提供了重要临床证据。

## 1 病例资料

患者男，36岁，于2023年1月因“右侧胸痛伴咳嗽半月”就诊于当地医院。胸部电子计算机断层扫描（computed tomography, CT）发现右肺多发结节，并伴有右侧胸腔积液。行胸水细胞学检测见部分间皮细胞轻度核异质改变，2023年1月29日行胸腔镜检查示壁层胸膜较大肿块及大量大小不等结节分布，脏层胸膜、膈胸膜大量结节分布，无法手术切除。患者于壁层胸膜处取活检，病理考虑恶性肿瘤。后患者就诊于华中科技大学同济医学院附属协和医院肿瘤中心，美国东部肿瘤协作组体能状态（Eastern Cooperative Oncology Group performance status, ECOG PS）评分为1分，无吸烟史，病理会诊为*ALK*阳性的恶性肿瘤，倾向于EIMS。免疫组织化学结果示：肿瘤细胞ALK呈阳性，CK-pan、细胞角蛋白5/6（cytokeratin 5/6, CK5/6）、甲状腺转录因子1（thyroid transcription factor-1, TTF-1）、Claudin-4、Calretinin、D2-40、S100、SMA、CD45、CD30、CD34、信号转导因子和转录活化因子6（signal transducer and activator of transcription 6, STAT6）、ERG、SS18-SSX、MyoD1、Myogenin、CgA、Syn、MelanA、人类黑色素瘤黑色素抗体-45（human melanoma black 45, HMB45）呈阴性，CD99呈弱阳性。进一步完善组织学下一代测序（next-generation sequencing, NGS）提示*TPM3-ALK*融合，突变峰度为34.97%。胸部增强CT显示右侧胸膜明显增厚、局部呈结节状/团块状，右肺上叶结节影，与邻近团块状增厚胸膜分界不清，整体范围约为99 mm×66 mm，增强扫描强化不均，考虑为肿瘤性病变（图1A）。正电子发射型计算机断层显像（positron emission tomography/CT, PET/CT）检查提示右肺上叶见不规则软组织结节影，大小约为2.8 cm×1.9 cm，SUVmax为6.5；右侧胸膜多处不均匀增厚，局部呈结节/团块状，大者截面大小约8.4 cm×5.0 cm，代谢异常增高；左侧耻骨代谢局限增高灶；以上考虑为右肺上叶原发伴有胸膜和左侧耻骨转移（图1B、1C）。

**图 1:**
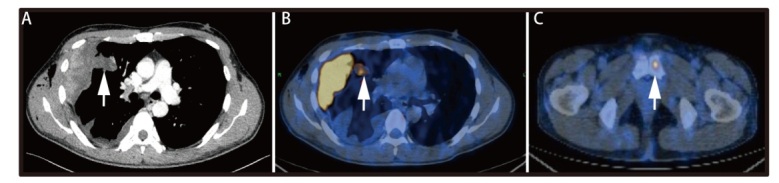
患者基线期影像学表现。A：增强CT示右肺病灶伴胸膜转移；B：PET/CT示右肺及胸膜转移病灶；C：PET/CT示左侧耻骨转移。

鉴于既往文献报告的第一代ALK-TKI克唑替尼治疗肺原发EIMS患者的PFS较短^[2,3]^，该患者于2023年2月15日接受了第二代ALK-TKI恩沙替尼的治疗，剂量为225 mg，每日一次。靶向治疗1个月后复查胸部CT增强显示肺部和胸膜病灶较前明显缩小，根据实体瘤疗效评价标准1.1（Response Evaluation Criteria in Solid Tumors 1.1, RESIST 1.1）的评估标准，实现了部分缓解（partial response, PR），且患者胸痛及咳嗽症状明显缓解。

为了进一步改善PFS，患者在2023年10月12日接受了肺部和胸膜残留病灶的适形调强放射治疗（intensity-modulated radiation therapy, IMRT），处方剂量为计划靶区（planning target volume, PTV）51 Gy/17 F， 2024年1月4日行左侧耻骨转移灶的IMRT，处方剂量为PTV 45 Gy/15 F。2025年7月1日患者复查颈胸腹部增强CT仍提示肺部和胸膜病变持续PR（图2）。脑部增强磁共振成像（magnetic resonance imaging, MRI）未见肿瘤转移征象。全身骨显像示左侧骶髂关节及左侧胫骨内侧髁骨质代谢轻度活跃灶，考虑骨及关节良性病变可能，建议随诊复查；上下颌骨骨质代谢轻度活跃灶，考虑多为局部炎症改变；其余部位骨骼骨质代谢未见明显异常，建议定期复查。截至2025年11月，患者的PFS已超过32个月，未发生明显的治疗相关不良反应。

**图 2:**
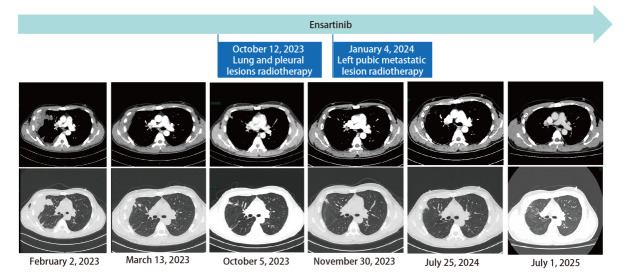
患者治疗经过及治疗期间影像学表现。基线期（2023-02-02），CT示右侧胸膜明显增厚、局部呈结节状/团块状，右肺上叶结节影，与邻近团块状增厚胸膜分界不清，整体范围约为99 mm×66 mm；靶向治疗后首次复查时间（2023-03-13），CT示右肺上叶及胸膜病灶较前明显缩小；靶向治疗8个月（2023-10-05），CT示以上病灶持续缩小；胸部病灶放疗后首次复查时间（2023-11-30），CT示右侧胸膜增厚、多发结节，右肺上叶结节大小约11 mm×7 mm，较前相仿；靶向治疗近18个月（2024-07-25），CT示右肺上叶及右侧胸膜病灶较前略缩小；末次复查时间（2025-07-01），CT示右肺上叶及胸膜病灶持续稳定。

该病例报道已取得患者知情同意并得到华中科技大学同济医学院附属协和医院医学伦理委员会批准（伦审字0533号）。

## 2 讨论

EIMS是一种间叶来源的恶性肿瘤，通常发生在成年男性的腹膜和肠系膜^[4,6,7]^。目前EIMS的最佳治疗模式仍未确定，根治性手术是早中期IMT的标准治疗手段，但对于复发性或转移性IMT目前没有标准治疗手段。克唑替尼、色瑞替尼和阿来替尼等第一、二代ALK-TKIs已显示对*ALK*融合突变的晚期EIMS有效，但报告的PFS大都不尽人意^[3,4,7]^。ALK-TKIs在非小细胞肺癌（non-small cell lung cancer, NSCLC）中的研究证实第二代ALK-TKIs恩沙替尼疗效优于第一代ALK-TKIs克唑替尼^[8]^。此外，荟萃分析^[9]^显示恩沙替尼对比其他ALK-TKIs，在亚裔人群中取得了最长的PFS。本例经一线恩沙替尼治疗后显示出超过32个月的持续缓解，目前病灶仍良好控制，预期PFS将更长。

放疗主要是利用射线对肿瘤细胞进行杀伤，延缓靶向耐药，靶向治疗则可通过抑制特异性信号通路增强放疗敏感性，两者联合可以发挥协同增敏效应^[10]^。既往小样本研究^[11]^发现在NSCLC中ALK-TKIs靶向治疗联合局部放疗可获得生存获益。第一代ALK-TKIs克唑替尼靶向治疗期间出现颅外寡进展后联合局部放疗能够显著延长患者中位总生存期（overall survival, OS）。另外一项II期研究^[12]^提示接受克唑替尼靶向治疗3个月后，针对残留病灶进行局部放疗PFS和OS均可获益。肺原发EIMS发生率极低，仅有少量病案报道^[2,3,13,14]^，且即使接受了第二代ALK-TKIs治疗，一线PFS仍不理想。本例患者的PFS远超既往病例数据，这种优势可能源于恩沙替尼治疗有效后联合原发残留和转移病灶的局部放疗产生的协同效应。靶向治疗结合局部放疗的联合治疗模式为肺原发EIMS的患者提供了新选择，但对起源于其他部位并伴有广泛转移的EIMS证据有限。

既往报道^[1]^的大多数EIMS病例均表现出*ALK*基因融合突变，常见的*ALK*融合伴侣基因包括*RANBP2*、*RRBP1*、*EML4*、*TPM3*和*TPM4*等。本病例的NGS检测到了*TPM3-ALK*融合突变体，也是首次尝试对该类融合变体使用第二代ALK-TKIs恩沙替尼的疗效探索，并联合局部放疗，最终取得良好的生存获益。若出现疾病进展，建议患者在条件允许的情况下再次行组织活检和基因检测，与*ALK*阳性的NSCLC相比，EIMS靶向治疗的耐药机制尚不清楚，需要更多的临床试验数据支持。

此外，除了*ALK*融合基因，临床需要进一步的研究来阐明EIMS的分子驱动因素并探索新的治疗靶点。有研究^[15]^发现了免疫检查点蛋白程序性细胞死亡配体1（programmed cell death ligand 1, PD-L1）和CD30在EIMS组织中的表达，提示了免疫检查点抑制剂和CD30单克隆抗体在EIMS中的潜在作用。

综上所述，本文总结了1例晚期肺原发EIMS患者的治疗经过，在遵循基于分子分型的靶向治疗原则上，探索了联合残留病灶及转移灶放疗在该疾病中的疗效，为该疾病的治疗模式提供初步证据，未来需开展相关临床研究来证实靶向联合放疗在EIMS中的有效性和安全性。
